# From Protein Building Blocks to Functional Materials

**DOI:** 10.1021/acsnano.0c08510

**Published:** 2021-03-24

**Authors:** Yi Shen, Aviad Levin, Ayaka Kamada, Zenon Toprakcioglu, Marc Rodriguez-Garcia, Yufan Xu, Tuomas P. J. Knowles

**Affiliations:** † Centre for Misfolding Diseases, Yusuf Hamied Department of Chemistry, University of Cambridge, Cambridge CB2 1EW, U.K.; ‡ School of Chemical and Biomolecular Engineering, The University of Sydney, 2006 Sydney, New South Wales, Australia; § Xampla, the BioInnovation Building, 25 Cambridge Science Park Road, Cambridge CB4 0FW, U.K.; ∥ Cavendish Laboratory, University of Cambridge, Cambridge CB3 0HE, U.K.

**Keywords:** protein, biomaterial, gel, fiber, film, microfluidic, self-assembly, drug delivery, condensate

## Abstract

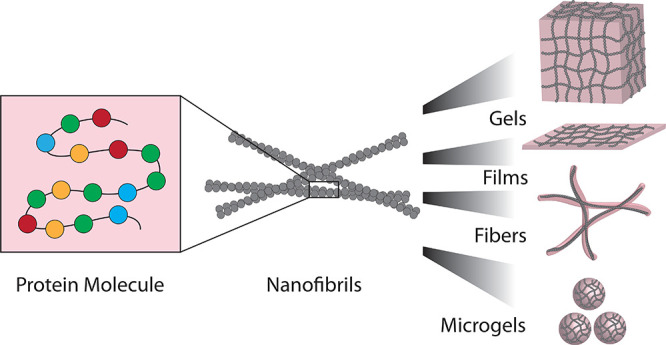

Proteins are the fundamental building blocks for high-performance materials in nature. Such materials fulfill structural roles, as in the case of silk and collagen, and can generate active structures including the cytoskeleton. Attention is increasingly turning to this versatile class of molecules for the synthesis of next-generation green functional materials for a range of applications. Protein nanofibrils are a fundamental supramolecular unit from which many macroscopic protein materials are formed. In this Review, we focus on the multiscale assembly of such protein nanofibrils formed from naturally occurring proteins into new supramolecular architectures and discuss how they can form the basis of material systems ranging from bulk gels, films, fibers, micro/nanogels, condensates, and active materials. We review current and emerging approaches to process and assemble these building blocks in a manner which is different to their natural evolutionarily selected role but allows the generation of tailored functionality, with a focus on microfluidic approaches. We finally discuss opportunities and challenges for this class of materials, including applications that can be involved in this material system which consists of fully natural, biocompatible, and biodegradable feedstocks yet has the potential to generate materials with performance and versatility rivalling that of the best synthetic polymers.

## Introduction

**Figure 1:**
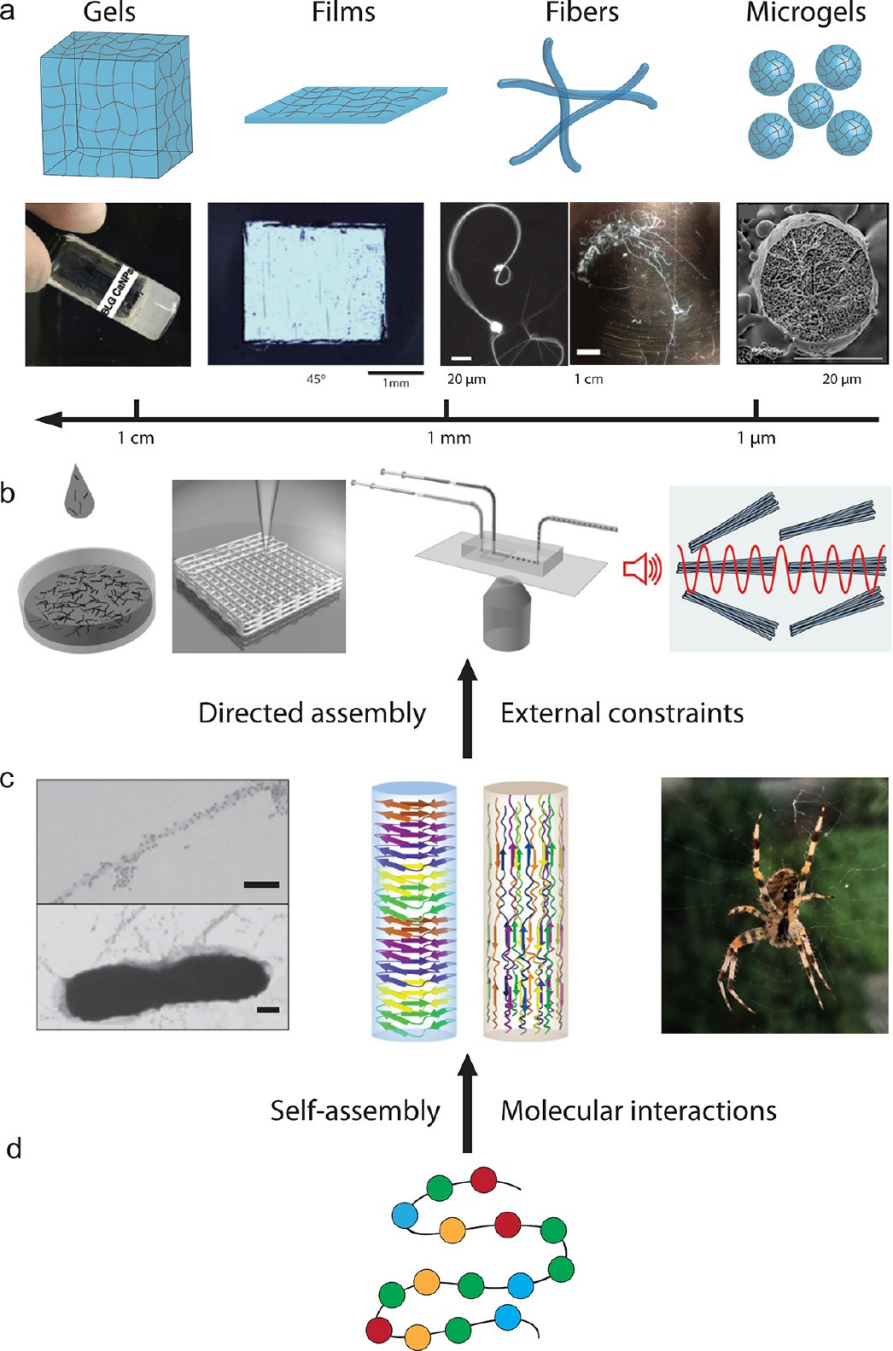
Assembly of proteins from the molecular level to functional materials. (a) Protein molecules can form bulk gels, films, fibers, and microgels.^2,5,38−40^ The examples represent BLG fibrillar hydrogel,^2^ lysozyme fibrillar film,^5^ a fiber formed from fused in sarcoma (FUS) protein condensates,^38^ whey protein fibrillar fibers,^39^ and cyro-scanning electron microscopy (SEM) image of a lysozyme fibrillar microgel.^40^ The scale bars are 1 mm, 20 μm, 1 cm, and 20 μm, respectively. The figure is adapted with permissions from ref (2), Copyright 2017 Wiley-VCH; ref (5), ref (38), Copyright 2010 and 2020, Springer Nature; ref (39); ref (40), Copyright 2015, American Chemical Society. (b) Methods that can direct the assembly of the building blocks into materials. The examples include drop casting, 3D printing,^41^ microfluidics, and ultrasonication.^16^ The figure is adapted with permissions from ref (41), Copyright 2008, Wiley-VCH; ref (16), Copyright 2019, Springer Nature. (c) Nanoscale fibrils containing antiparallel and parallel β-sheets are the building blocks of the natural protein materials.^42,43^ The figure is adapted with permissions from ref (42), Copyright 2014, Springer Nature; ref (43), Copyright 2014, Wiley-VCH. (d) Nanofibrils’ formation is a result of protein molecule self-assembly.

Biomaterials generated from natural components, such as proteins and peptides, have attracted much attention because of their biocompatibility and sustainability.^1−3^ Capitalizing on their naturally evolved capacity to assemble into functional complexes under biocompatible conditions, proteins can be engineered to generate biomaterials exhibiting a wide range of macroscopic structures, such as bulk gels, films, fibers, microgels, and active materials with the scale spans from microns to centimeters (Figure 1a). Further expanding on this capability, composite protein materials have been recently developed by incorporating other polymers, inorganic nanoparticles, and dye molecules, which introduced additional functions to these biomaterials.^4−6^ The applications in drug/nutrition delivery,^7−10^ antitumor therapy,^11−13^ and cell growth scaffold^14,15^ have a huge impact on biomedical advances and food/pharmaceutical industries. A commonly used strategy in nature to form functional protein materials is a two-step process, where the nanoscale structure of the material is determined by molecular recognition and self-assembly and the micro to macro scale structure, including high-order-oriented organization is imposed through external constraints (Figure 1b–d).^16,17^ This strategy is used, for example, in the spinning of silk, where the supramolecular β-sheet units are formed through self-assembly and the filament morphology is a result of the spinning process.^18,19^ This approach represents a combination of top down and bottom up approaches to shape the final material. A particularly versatile strategy that has allowed this natural multistep process to be implemented in an artificial setting is to use self-assembling protein nanofibrils as the building blocks of functional materials.

These nanoscale building blocks can be formed from a wide variety of polypeptide sequences and are typically stabilized by extended supramolecular β-sheet networks through self-assembly (Figure 1c,d). One of the best known natural examples of self-assembled protein biomaterials found in nature is spider silk, composed of silk nanofibrils exhibiting parallel and antiparallel β-sheets in their secondary structure. Such nanofibrils have been shown to be generated through a natural spinning process by spiders or silkworms and have been well characterized in recent years because of their extraordinary mechanical properties. Similarly, amyloid nanofibrils have initially been studied because of their role in human neurodegenerative disorders, such as Alzheimer’s and Parkinson’s diseases.^20^ Yet, such amyloid fibrils generated through the aggregation of a wide range of proteins have further been discovered in many organisms where they have biological functional roles, such as curli in *E. coli*.^21−24^ Recently, there is a marked rising interest in assembling protein into microscopic functional biomaterials by using the nanofibrils as building blocks.^25−30^

The multiscale construction of the nano building blocks, such as confinement, orientation, and helicoildal stacking are significant to the properties and functions of the resulting materials. In spider silk fibers, nanoconfinement of the β-sheets with an optimal size 2–4 nm leads to high strength, stiffness, and toughness.^19,31^ Moreover, oriented alignment of anisotropic fibers by external fields also result in materials with hierarchical structures and excellent mechanical properties.^16^ Additionally, the 3D helicoildal stacking, commonly found in chitin nanofibril-based materials, has an important role in the final mechanical and optical properties.^19,32,33^

Furthermore, both naturally evolved and artificial nanofibrils can be applied for the generation of mechanical force. As a result of misfolding, the individual polypeptides constituting an amyloid fibril form predominantly β-sheet rich structure through noncovalent interaction. These interactions give rise to stacking of proteins and peptides into ordered fibrils with a diameter of several nanometers and lengths in the micron scale. Thus, amyloid fibrils can be regarded as biopolymers with common structural and mechanical properties rather than through their specific chemical composition. Amyloid fibrils can form spontaneously from a wide range of chemically simple building blocks, and their structural and mechanical properties are relatively insensitive to the protein’s specific amino acid sequence.^34^ The cross β-sheet core structure of amyloid fibrils has been found to be very rigid and confers superior mechanical properties on the structures formed through self-assembly. Thus, amyloid fibrils can exhibit a Young’s modulus similar to that of silk and an ultimate strength similar to steel.^8,35^ As such, amyloid fibrils constitute promising building blocks for bioinspired materials. For example, fibrils formed by β-lactoglobulin (BLG) found in milk, have been used alone or together with nanocomposites to generate functional biomaterials and exhibit a diverse set of applications in terms of human nutrition,^4^ biosensing properties,^36^ conductivity,^37^ mechanical strength,^6^ and the ability to be applied for water purification^3^ in either liquid or solid form. These examples suggest that protein-based fibrils with the flexibility to be incorporated with various compounds, have a huge potential for the generation of multifunctional biomaterials.

There are many approaches can be used to apply external constraints to direct the assembly of protein nanofibrils, forming macroscopic materials, such as drop casting,^5^ 3D printing,^44−46^ microfluidics^38,47−49^ for molding and shaping and ultrasonication for orientation^50^ (Figure 1b). Among them, microfluidic techniques have been used as a platform for sample analysis,^51−54^ microreactor,^55,56^ and encapsulation^57^ with the advantages of limited sample consumption, high throughput, short experimental time, and being fully customizable with respect to conventional bulk experiments. It is an ideal tool to apply shear, control the environment, and provide confined geometries. Using these techniques, protein fibers can be generated using contracting flow to control the orientation of the subunits. The obtained fibers had high strength and toughness because of the alignment of the nanofibrils.^58^ Similarly, microfluidics allow the production of microgels by encapsulating the protein in segmental flow and inducing nanofibril formation inside the droplets.^53,9,59,60^ Such microfluidic platforms further provide the ability and the flexibility to form multilayer core–shell microgels.^9,61,62^ In this Review, we explore the development of microfluidic techniques to guide the assembly of nanoscale building blocks into a wide range of structures. We further provide information on how the formation of such structures allows the tailoring of certain properties and functions of the generated materials and how these can be precisely controlled using microfluidic base approaches.

## Fibrous Protein Gels

Self-assembled nanofibrils generated from proteins and peptides can be used to generate hydrogels and aerogels. Hydrogels formed through physical gelation or chemical cross-linking have the capacity of holding a large amount of water in their polymeric network. By carefully removing the water without disrupting the self-assembled protein network, hydrogels can be transformed to aerogels with ultralight weight, high porosity, and ultralow thermal conductivity.^63−65^ Recently regenerated silk fibroin was also used as a thermoplastic at elevated temperature and pressure for materials with tunable mechanical properties.^66^ Because of the inherent biocompatibility and biodegradability of the building blocks, nanostructured protein hydrogels and aerogels exhibit a potential to be applied in numerous applications especially in food and biomedical fields.

The fibrillar hydrogels can be generated from a wide range of proteins, such as β-lactoglobulin,^2,6,67,68^ lysozyme,^5^ soy,^69^ silk proteins,^70,71^ elastin,^72,73^ collagen,^15,46^ and bovine serum albumin.^74^ In the previous study, the sol–gel transition of β-lactoglobulin fibrils at different protein concentration and ionic strength was investigated and provided the fundamentals of using amyloid fibrils as building blocks for biomaterials.^67^ Furthermore, the hydrogels derived from plant and milk proteins were produced and characterized and shed light on the applications of fibrillar hydrogels in food systems.^68,75−77^

**Figure 2:**
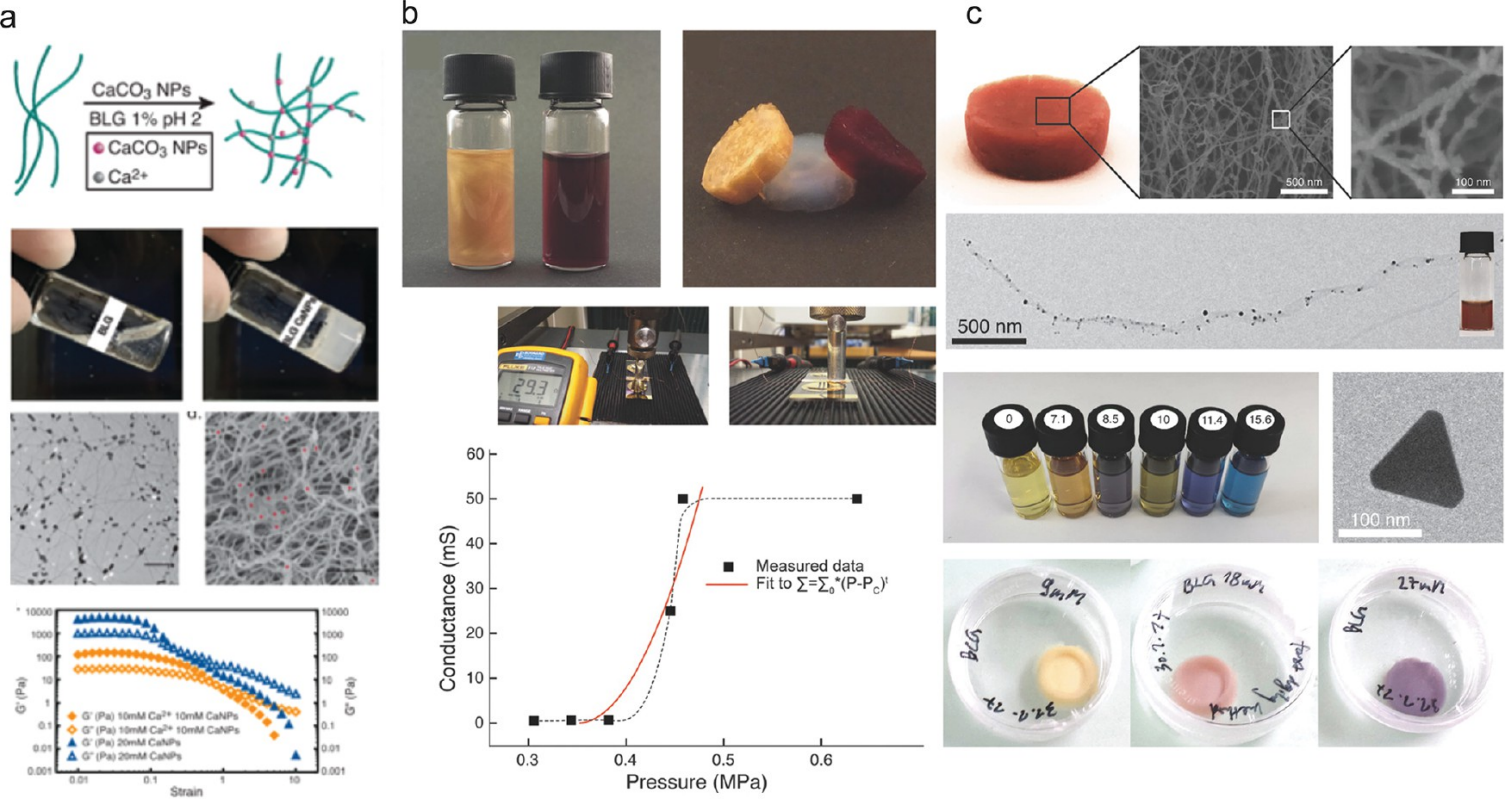
Hydrogels and aerogels generated by protein nanofibrils and nanoparticles (NPs). (a) Gels made of β-lactoglobulin (BLG) fibrils and CaNPs with both Ca^2+^ and CaNPs working as cross-linkers to stabilize the network. The figure is adapted with permission from ref (2), Copyright 2017, Wiley-VCH. (b) Aerogels formed by BLG fibrils and Au crystals and AuNPs. Amyloid aerogel with imbedded gold crystals showed increasing conductivity when an increasing pressure is applied.^6,78^ (c) Aerogels made of BLG fibrils and AgNPs showed optical properties.^78^ Figures adapted with permissions from ref (6), Copyright 2015, Wiley-VCH; ref (7), Copyright 2017, Wiley-VCH.

Recently, hybrid fibrillar hydrogels have been developed by mixing protein fibrils with other compounds, such as nanocellulose^79^ and inorganic nanoparticles^2,6,78,80^ for desired mechanical properties and versatile functions. In this manner, the incorporation of calcium nanoparticles (CaNPs) into β-lactoglobulin fibrils allowed establishing and stabilizing the fibrillar network. The CaNPs worked as cross-linkers with multiple binding spots, connecting protein fibrils to enhance the strength of the gel network. The resulting hydrogels showed a 2 orders of magnitude increase in gel strength compared with previous studies on material properties of BLG gels. This system further exhibited a self-healing property demonstrated by the recovery of the storage modulus (Figure 2a).^81^ Similarly, hydrogels and aerogels have been formed with gold/silver nanoparticles and crystals decorated on the β-lactoglobulin fibrils in the network. Gold nanoparticles and crystals were formed directly on the BLG fibrils by reducing the gold salt. The aerogels derived from the hydrogels presented electrical conductivities, pressure sensing, and possibilities for plasmonic sensing (Figure 2b).^82^ Depending on the pH value of the gelling conditions, the final hydrogels with silver nanoparticles exhibit tunable optical properties, with wavelength absorbance ranging from orange to purple (Figure 2c).^83^

Bulk gels with nanofibrils as a network have been studied for fundamental science and show great potential in many applications, especially when incorporated with inorganic nanocomposites. On this basis, further processing steps can be applied to assemble and tailor the materials with desired sizes, shapes, and structures.

## Films, Coatings, and Interfaces

Proteins and their self-assemblies have been used to fabricate 2D films with a wide range of functionalities in many applications because of their native properties^5,43,84^ and synergetic effects when combined with other nanomaterials.^3,36,37,85^ Processing methods, such as drop casting,^5,85^ vacuum filtration,^3,36,37,43^ and interface collection have been performed to assemble these protein films to obtain desired mechanical, optical, chemical properties.

**Figure 3:**
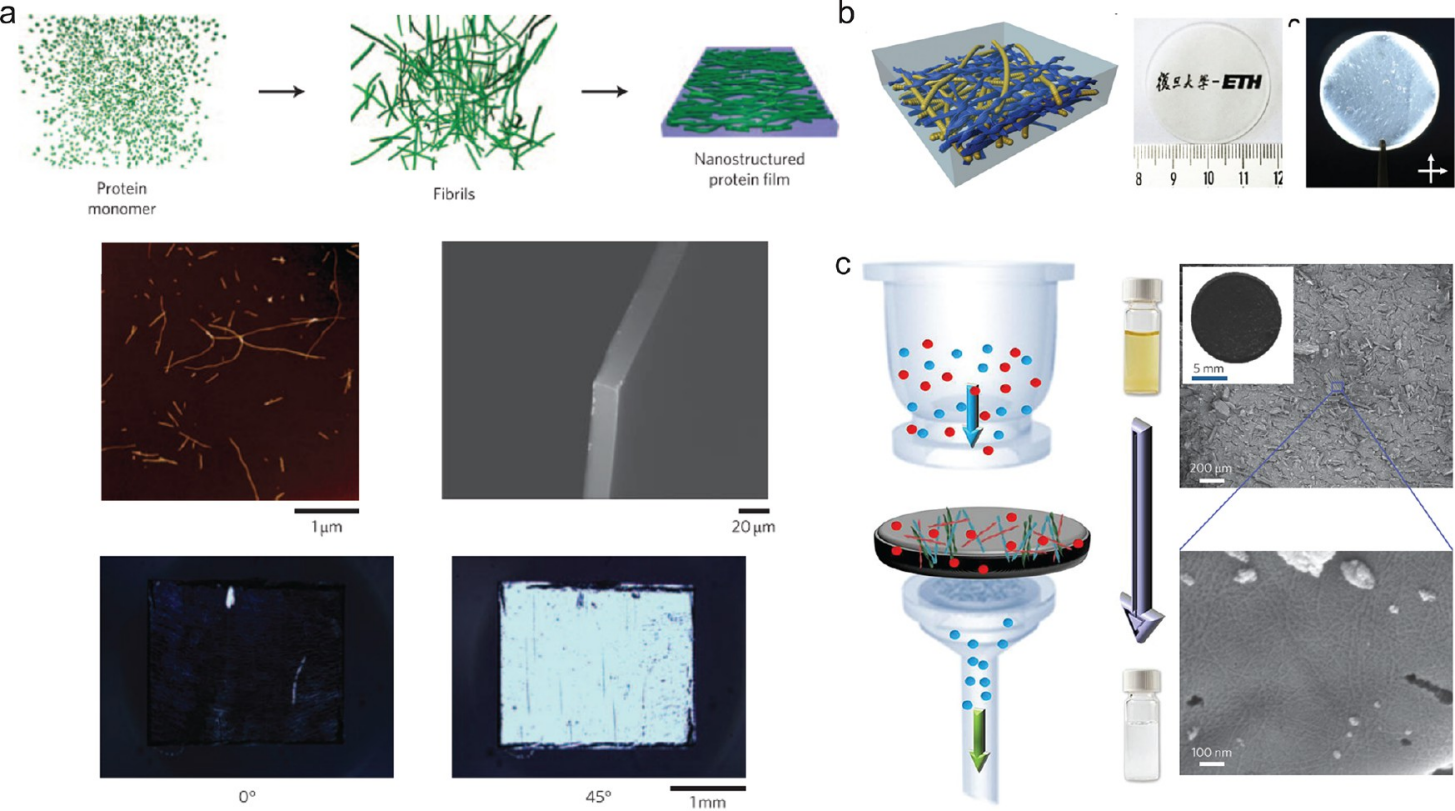
Protein films and protein hybrid films. (a) Lysozyme was used to form the nanofibrils structured 2D film (top). This free-standing film with ordered nanostructures showed peaked birefringence signal at 45 deg under cross-polarized microscopy (bottom).^5^ The figure is adapted with permission from ref (5), Copyright 2010, Springer Nature. (b) Films made of silk and BLG fibrils showed transparent optical property and birefringence signal under cross-polarized microscopy. The figure is adapted with permission from ref (43), Copyright, 2014 Wiley-VCH. (c) Schematic illustration of purification process by amyloid–carbon film (left), and color change of Na_2_PdCl_4_ solution after filtration (middle). SEM image showing general and detailed structure of the amyloid-carbon film (right).^3^ The figure is adapted with permission from ref (3), Copyright 2016, Springer Nature.

Lysozyme and β-lactoglobulin are proteins that can be isolated from animal-derived food products (such as eggs and milk, correspondingly) and are considered to be safe material sources with low cost and high biocompatibility.^86^ Both proteins can form antiparallel β-sheet rich amyloid fibrils upon hydrolysis and incubation. This feature has been applied in the use of lysozyme amyloid fibrils to generate free-standing films with great mechanical and optical properties by using a solvent-casting method. It has thus been demonstrated that the hierarchical self-assembling proteins aligned unstructured fluorophores, resulting in the formation of protein films with polarized fluorescence (Figure 3a).

Silk fibers containing nanofibrils rich in parallel β-sheets exhibit excellent mechanical properties.^87^ Silk fibrils (SF) from fibroin and amyloid fibrils (AF) from BLG have been shown to generate films by concentrating the solution through vacuum filtration. The mechanical properties of the films produced can be tuned by the ratio of SF:AF because of the orientation of b-strands in the fibrils (Figure 3b). With the same total amount of protein, the films show increased toughness with more SF and higher tensile moduli with more AF. Such films have further exhibited magnetic properties through their decoration with iron nanoparticles onto the fibrils directly.

### Organic–Inorganic Hybrid Films

Proteins are inherently highly biocompatible and can be further equipped with inorganic components. Combining protein-based building blocks with inorganic nanomaterials can increase the chemical and physical space of their properties and allow a wide variety of applications.^86^ Formation of membranes by pressing BLG amyloid fibrils and active carbon through a vacuum pump has been recently demonstrated, resulting in films that can selectively absorb heavy metal ions from solutions and reduce them into metallic form (Figure 3c). The technology is a breakthrough of global water pollution and treatment.^3^ In another study, α-synuclein and gold nanoparticles have further been employed for monolayer film fabrication. These free-standing films were formed and collected by absorbing the αS-AuNPs monolayer onto thin polycarbonate substrate first and then dissolving the substrate to obtain the films. The authors demonstrated the flexibility and scalability of the films, which can be important in development of nanodevices and biosensors.^85^

## Macroscopic Fibers and Yarns

### Natural Spinning Process

Silk fibrils biosynthesised by spiders and silkworm are one representative example of the mechanisms involved in protein nanofibrils assembly into 1D materials. The production of silk fibers takes place in the spinning duct of spiders and silkworms, where protein molecules are confined into a long micron-scale channel. While silk fibers are processed, the protein solution is pushed through to the end of the duct and exposed to a gradient of ions and pH, as well as hydrodynamic shear generated by the contraction of the spinning duct.^88,89^ Such shear force not only promotes the self-assembly of proteins into fibrils but also assists the alignment of proteins by stretching the molecules, resulting in well-ordered fibrillar networks in the fiber, which is key for the mechanical performance of silk thread.^48,90−94^ This spinning process does not require harsh conditions and is achieved at room temperature with low energy input. Therefore, mimicking this strategy can provide materials with excellent mechanical properties as well as low-cost, energy efficient production of synthetic fibril-based fibrer.^95−98^

### Engineering Artificial Fibers

#### Bulk Self-Assembly

The ability of proteins and peptides to self-assemble into ordered structures is utilized to produce microscale fibers. For example, a peptide whose sequence was derived from suberin proteins of squid sucker ring teeth was shown to self-assemble into microscale fibers.^99^ The fibers contained well-oriented nanofibers formed by the peptide, resulting in excellent mechanical properties with Young’s Modulus of 7.7 GPa. Yet, while their structure was controlled during assembly, the duration for formation of ordered structures may extend up to 2 weeks. The slow assembly kinetics is also present in other systems reported to form microfibers through self-assembly. One such example can be seen in a mixture of wheat gluten proteins and peptides that takes 20 days to be assembled into microscale fibers.^100^ In addition, the self-assembly is a stochastic process, resulting in erratic diameters and morphologies. The length of fibers is limited to a few micrometers, which is not suitable for many applications, where spinning of continuous long fibers is preferred.

#### Bulk Spinning Processes

The most commonly used technique in the spinning of protein-based fibers is the wet-spinning process, where a protein solution is extruded into a coagulation bath and forms micron-sized continuous fibers.^101−104^ For example, methanol has been used extensively as a coagulation bath for wet-spinning of regenerated silk fibroin.^105^ Methanol promotes the assembly of silk fibroins into ordered nanofibrils and thus generates a hydrogel fiber composed of silk nanofibrils. While the wet-spinning method induces a shear force by extrusion during assembly, the shear-induced alignment of structure is often not sufficient during wet-spinning, and thus, the formed fibers often exhibit poor mechanical properties. Therefore, the automatic drawing process and subsequent stretching of fibers have been combined with wet-spinning techniques, leading to an improved alignment of fibrils by poststretching.^106−109^ These efforts successfully improve the mechanical properties of the formed fibers, resulting in performance close or even superior to that of natural silkworm silk.

**Figure 4:**
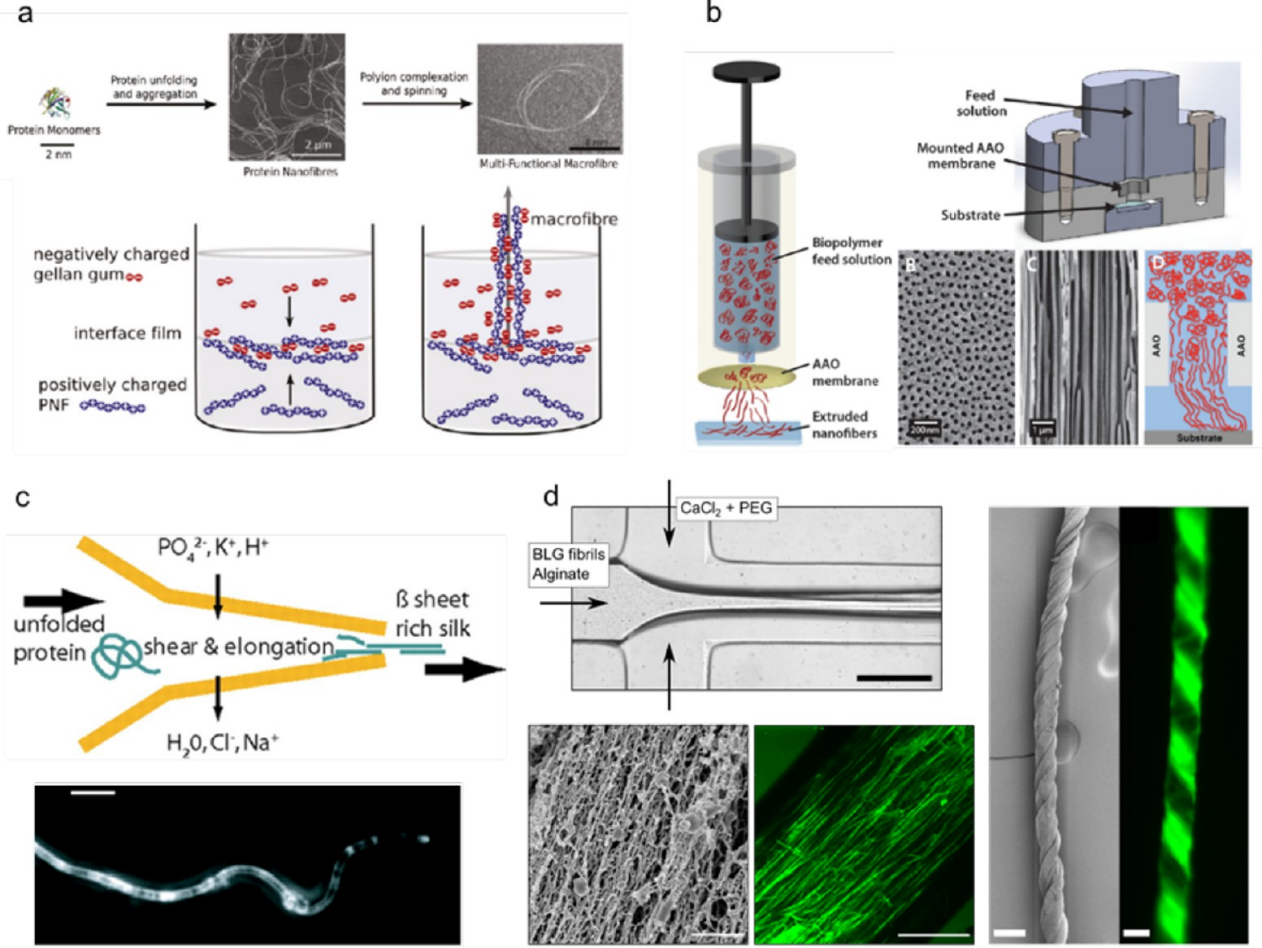
Assembly of protein nanofibrils into microfibers. (a) Amyloid fibrils from lysozyme assembled into microfibers using the wet-spinning technique. An oppositely charged polysaccharide was used to generate hydrogel fibers. The figure is adapted with permission from ref (27), Copyright 2011, American Chemical Society. (b) Fibronectin fibers were generated by extrusion of proteins through nanopore membrane. The proteins undergo conformational changes during extrusion. The figure is adapted with permissions under Creative Commons CC BY license from ref (120), Copyright 2016, Oxford University Press (left); with permission from ref (119), Copyright 2015, American Chemical Society (right). (c) Microfluidic spinning of recombinant silk protein resulted in aligned and β-sheet rich fibers. Cross-polarized optical microscopy image shows birefringence in the microfiber (bottom). The figure is adapted with permission from ref (122), Copyright 2008, National Academy of Sciences, U.S.A. (d) Microfluidic device was used to generate fibers with aligned β-lactoglobulin nanofibrils. Left top panel shows the optical image of the microfluidic channel. Left bottom panels show the SEM image of the hydrogel fiber (left) and the confocal image of a fiber stained with Thioflavin T to visualize the protein fibrils (right). Right panels show the SEM image (left) and fluorescent image (right) of yarn-like fibers generated through microfluidic spinning. This figure is adapted with permission from, ref (48), Copyright 2019, Wiley-VCH.

Solidification of fibers in wet-spinning can also be achieved by using oppositely charged polyelectrolytes. In the previous study, the wet-spinning technique was used to create microfibers from positively charged lysozyme fibrils with negatively charged polysaccharides, gellan gum (Figure 4a).^27^ Another study generated fibers by extruding Fmoc-diphenylalanine peptide solution into oppositely charged polymer solution, cationic polyacrylamide.^110^ The oppositely charged polymer promotes the formation of amyloid-like nanofibers from peptides, resulting in the creation of microfibers composed of β-sheet rich nanofibrils.

While in the above examples the fibers are solidified at the liquid interface, fibers can also be solidified by solvent evaporation, such as dry-spinning^111−113^ and electrospinning.^114−118^ Recently, fibronectin, actin, and myosin fibers have been generated by extrusion of the solution through a nonporous membrane in air (Figure 4b).^119,120^ Interestingly, these proteins undergo conformational changes during the extrusion, similar to the formation of silk fibers in the natural spinning process. In the electrospinning process, a strong electric field is applied to the protein solution for extrusion. This method generally produces fibers with nanoscale diameter, which enables drying them instantly while the proteins are traveling in the air from the nozzle to the collection mat. However, it should be considered that the electrospinning process is quite different from the natural spinning process, where hierarchical structures are formed through slow and controlled assembly.^121^ Once the fibers are formed in the electrospinning process, the protein solution is dried within milliseconds, which prevents the proteins from assembling into higher-order structures.

#### Microfluidic Spinning

Microfluidic spinning is a powerful way to assemble nanoscale building blocks into fibers in a controlled manner. Microfluidic spinning enables the confinement of the flow in a micron-scale channel, and thus, the resulting laminar flow profile allows exerting control over the chemical environment and hydrodynamic forces applied to the proteins, in a similar manner to the natural spinning process.^123−127^ In particular, the hydrodynamic shear in microfluidic channels has been utilized intensively to control the orientation of building blocks composing the nanofibrillar structure, including metal nanowires, carbon nanotubes (CNT), and cellulose nanofibrils.^19,39,128−131^ This is achieved by use of an extension flow, where the flow is narrowed geometrically and thus causes the stretch of molecules along the direction of flow (Figure 4c). In recent studies, such shear force was utilized to induce the alignment of protein nanofibrils.^48^ The orientation of β-lactoglobulin fibrils was precisely controlled by the flow rate, resulting in highly aligned nanofibrils and enhanced mechanical performance (Figure 4d).

Moreover, hydrodynamic shear was shown to regulate the protein secondary structure. One of the best known examples of shear-sensitive proteins is natural silk proteins.^9,132,133^ Shear force promotes the aggregation of proteins and eventually leads to the formation of β-sheet rich silk nanofibrils.^134^ Recently, microfluidic techniques have been used to study the assembly mechanism of silk recombinant protein eADF3/eADF4 (Figure 4c).^122^ While the self-assembly of recombinant silk proteins is quite different from one of the native silks, the results demonstrated that shear force is required for the successful generation of fibers. These studies suggest that shear force plays a key role in the assembly process of protein fibers, and thus, microfluidic spinning is a useful approach to generate fibers by controlling hydrodynamic forces precisely during the assembly.

## Hierarchical Structures and Micro/Nanogels

The production of biomaterial-based micron-scale emulsions for the storage and/or release of small molecules is of key relevance in the biomedical and biotechnological fields.^135^ Yet, systematically controlling the release kinetics of drug molecules for applications such as targeted therapy has remained challenging because of the high polydispersity exhibited by most conventional emulsification methods.^60^ The microfluidic techniques have contributed to the production of monodisperse droplets on a large scale, which in turn allowed the generation of peptide and protein-based materials.^47,136,9^ Microfluidic devices consisting of a 2D T-junction can readily be used for producing water-in-oil (w/o) or oil-in-water (o/w) emulsions, where control of the droplet size is well established and depends on the ratio of flow between the dispersed and continuous phases as well as on channel dimensions. Because of the relative ease of fabrication and high level of control over droplet monodispersity, conventional-PDMS based droplet microfluidics has emerged as a high-throughput method of generating hierarchical biomaterials for a range of applications in the context of encapsulating/releasing of small molecules for drug/gene delivery or for storage.^9,137^

### Microgels

**Figure 5:**
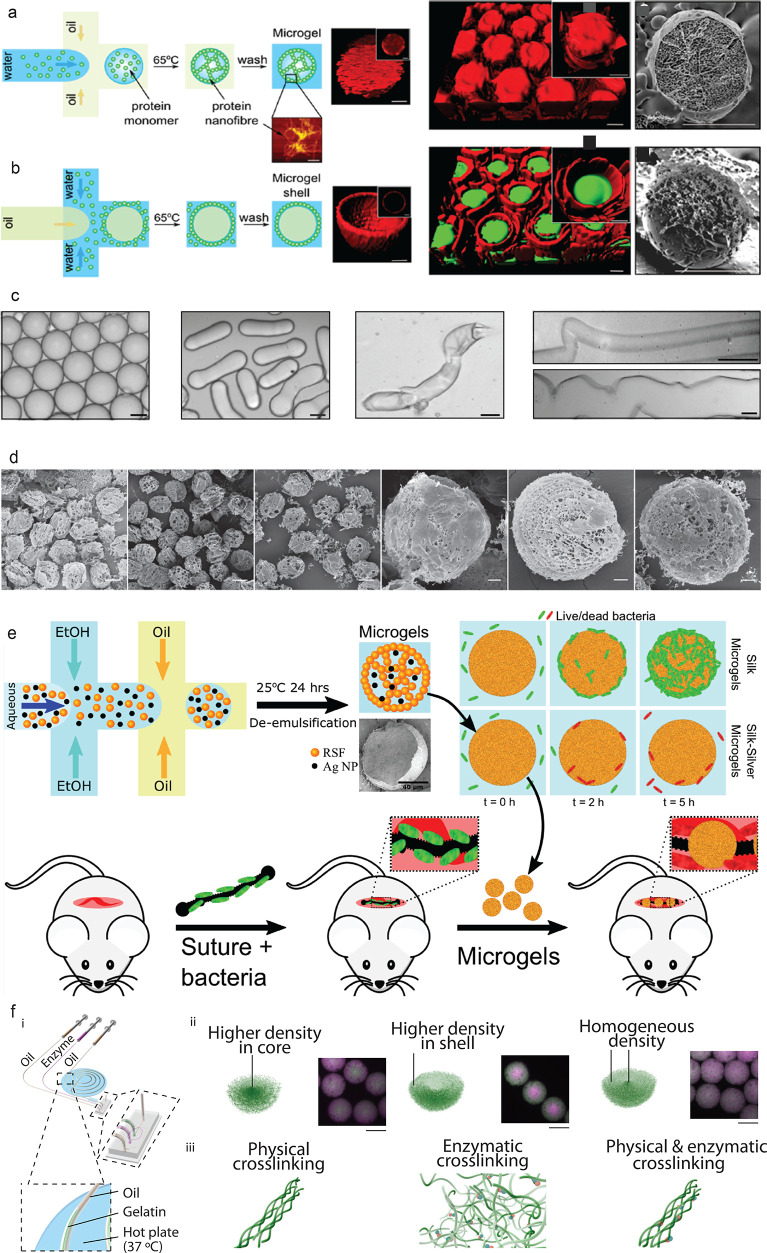
(a,b) Protein microcapsules. Schematic representation of forming water-in-oil lysozyme-based microgels and oil-in-water microgels, respectively. Middle and right panels depict confocal and cryo-SEM micrographs of the corresponding microgel systems. Figure adapted with permission from ref (40), Copyright 2015, American Chemical Society. (c) A range of native silk microgel morphologies can be formed on the basis of the solution flow rates applied. From left to right: spheres, cylinders, short fibers, and thin fibers. Scale bar, 5 μm. Figure adapted with permission under a Creative Commons CC BY license from ref (9), Copyright 2017, Springer Nature. (d) SEM micrographs of regenerated silk fibroin microgels formed by varying the ethanol content from 20 to 40%. The panels on the right are magnified micrographs of the corresponding images on the left panels. The scale bars are 50 and 10 μm from left to right. Reprinted with permission from ref (138), Copyright 2019, Wiley-VCH. (e) Schematic showing the production of the hybrid inorganic/organic microgels and their subsequent use as antibacterial agents for a surgical site on a murine model. Reprinted with permission from ref (139), Copyright 2020, American Chemical Society. (f) Spatially inhomogeneous gelatin microgels. i, Microfluidic setup with heating accessories. ii, Gelatin microgels with different radial density. Green and red (magenta) nanospheres were premixed in the gelatin and enzyme (transglutaminase) solutions, respectively. Scale bar, 100 μm. iii, Gelatin microgels through versatile cross-linking regimes. Figure adapted with permission under a Creative Commons CC BY license from ref (47), Copyright 2020, Wiley-VCH.

Previous studies demonstrated the formation of biomaterials by combining droplet microfluidics with protein self-assembly.^40^ Here, the generation of both w/o and o/w emulsions was achieved with the aqueous phase being composed of a monomeric protein solution. As can be seen from Figure 5a, initially w/o emulsions have been produced on-chip by having the aqueous solution intersect with fluorinated oil. Following droplet formation, the emulsion is collected and incubated at 65 °C in order to promote protein self-assembly, which results in the protein forming a fibrillar network within the droplet. The oil is then washed away, and the protein-based microgel is re-emulsified in an aqueous environment. Confocal microscopy, atomic force microscopy, and scanning electron microscopy have been employed in order to characterize the microgels, and it is clear from Figure 5a,b that a dense fibrillar network exists within the microgel. Additionally, o/w emulsions were prepared via the same protocol (Figure 5b), resulting in core–shell structures being formed. It was further found that the network and fibril formation within the microgels was pH dependent, with the lower pH microgels being more stable and having a denser network. In order to investigate the potential of these microgels for drug-delivery applications, small molecules have been encapsulated within the fibrillar network, and their release kinetics was monitored. The mechanism by which the molecules are released appears to follow a multistep process; in the first stage, unbound molecules are released into the surrounding aqueous environment. This is then followed by the release of the remaining trapped molecules into the solution. Furthermore, cell viability assays have been conducted and have indicated that the microgels were completely viable and nontoxic to the cells, suggesting that this material can be used for biological studies.

In more recent studies, native and regenerated silk fibroin (RSF) have been utilized to form microgels.^141^ As RSF is FDA approved, its potential in the pharmaceutical and drug-related fields has been extensively studied. The same experimental protocol for generating the microgels was followed; however, because of silks propensity to aggregate when subjected to a shear flow, different morphologies of microgels could be formed (Figure 5c). Depending on the flow rate, these structures ranged from spheres, all the way to large fibers, and they could be precisely controlled. Furthermore, the more stable RSF was used to form microgels where the release kinetics could be specifically tailored depending on how the microgel was formed (Figure 5d).^138^ In this study, ethanol was used to promote protein self-assembly by mixing it with the silk on-chip, and it was found that depending on whether the protein was surrounded by the ethanol or vice versa, microgel morphology was altered and consequently release kinetics of small molecules could be controlled more readily. Moreover, inorganic/organic hybrid microgels using RSF have been generated microfluidically (Figure 5e).^139^ By decorating the microgels with silver nanoparticles, these hybrid particles showed potent antimicrobial properties both *in vivo* and *in vitro* and bacterial eradication was demonstrated through a two-step mechanism of action. In contrast to conventional methods involving silver, these hybrid microgels were nonhemolytic and noncytotoxic toward mammalian cells, making them ideal for wound-related applications, as shown by use of a murine model, where a surgical site infection was treated using the inorganic/organic microgels. Additionally, native silk fibroin (NSF), which was directly extracted from silk worms, can form photonic material.^140^ It was demonstrated that NSF can be structured into microcapsules with tunable autofluorescent signal as optically active material.

Gelatin inherits certain peptide sequences and triple helices from collagen and has higher solubility at neutral pH, less immunogenicity, better formability, and printability than native collagen.^45,47,142−146^ It is significant to the applications in 3D cell-culture studies in regenerative medicine and the construction of disease models.^47,144,147,148^ By exploiting a simple microfluidic setup to control the temperature, gelatin microgels with a set of inhomogeneous structures can be formed (Figure 5f (i).^47,139,140^ It has been studied that a set of spatially inhomogeneous microgels can be manufactured by adjusting microfluidic mixing, chip geometries, as well as versatile cross-linking regimes (Figure 5f (ii);^47^ radial gradients can be achieved in theses microgels, including higher density near the cores, higher density near the shells, with homogeneous radial density as control studies.^47^ In addition, the spatially inhomogeneous microgels demonstrate distinctively different dissolution performances though enzymatic digestion.^47^ Furthermore, Janus microgels have been made in liquid–liquid phase separated microdroplets with a specific range of the volume ratio of gelatin to polyethylene glycol (PEG) solutions in oil droplets.^149^ Another method of fast production of Janus microgels has been described by the fusion of two protein (gelatin) droplets in a crowding agent (PEG solution).^150^ The gelatin-PEG all-aqueous systems have enormous potential in applications such as 3D cell culture, 3D printing, and temperature sensing.^150−152^

### Nanogels and Multiscale Gels

**Figure 6:**
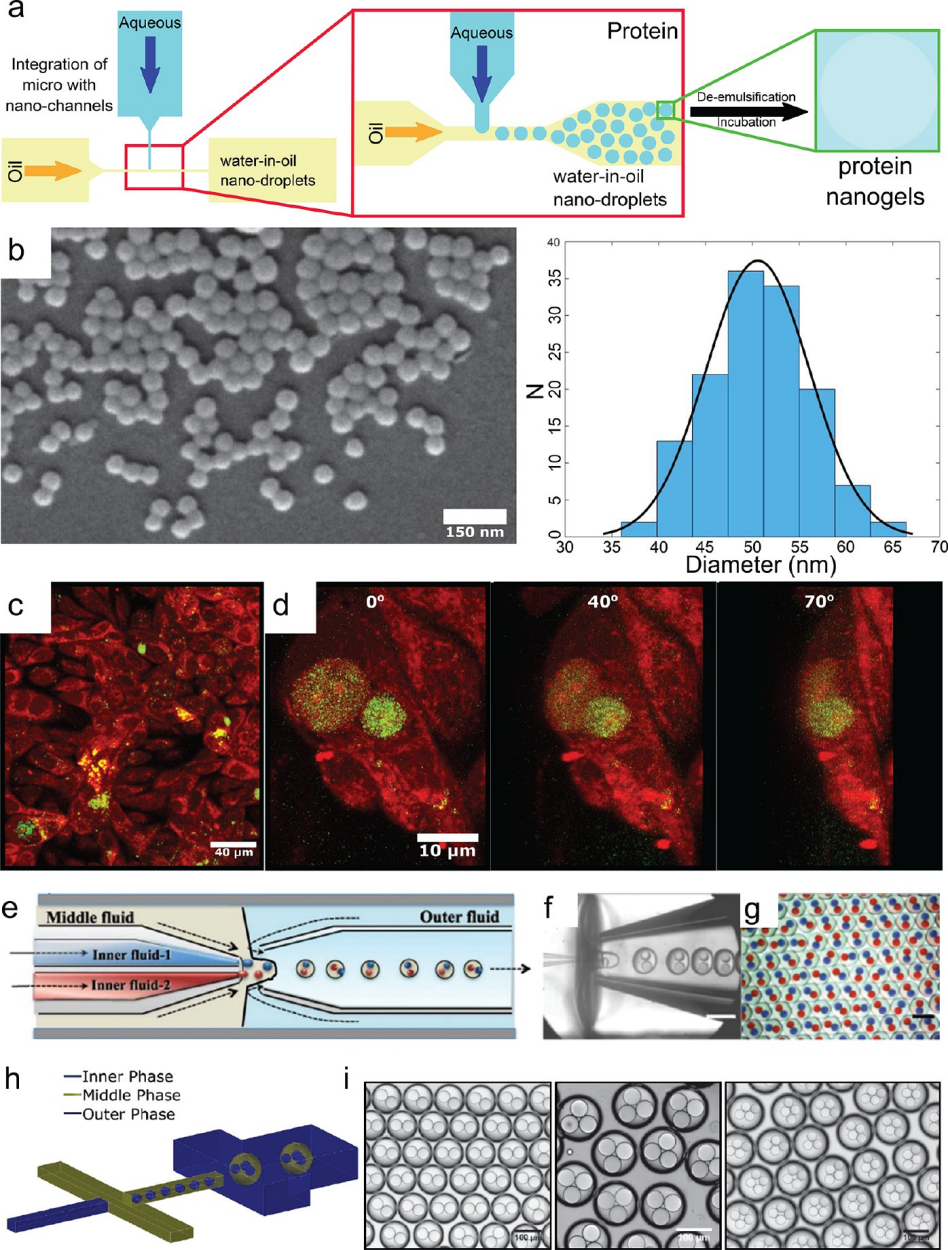
(a) Schematic representation of the hybrid nano/microfluidic device used to generate water-in-oil nanodroplets and their subsequent formation into nanogels. (b) SEM micrographs of the silk nanoparticles and the corresponding size distribution histogram. (c) Confocal microscopy images of ovarian cancer cells (red) in the presence of silk nanoparticles (green). (d) 3D reconstruction of a single cancer cell which was imaged at different angles with respect to the *z* axis in order to show that the nanoparticles have penetrated the membrane and are well within the cell. Figures a–d adapted with permission under a Creative Commons CC BY license from ref (49), Copyright 2020, American Association for the Advancement of Science. (e–g) Schematic diagram of the capillary device used to form hierarchical emulsions with two different types of aqueous droplets and corresponding brightfield microscopy images of these double emulsions. Figure adapted with permission from ref (153), Copyright 2012, Royal Society of Chemistry. (h) Schematic representation of the 3-D devices used to generate hierarchical emulsions. (i) Collected droplets consisting of two, three, or four internal droplets. (h, i) Figure adapted with permission from ref (154), Copyright 2017, American Chemical Society.

Moreover, nanosized channels have been integrated with microchannels, and subsequently, protein-containing nanodroplets (including RSF-based) have also been formed using a hybrid nano/microfluidic device (Figure 6a,b).^49^ Thus, RSF was used for the formation of both micron and submicron emulsions, where the dispersed phase was composed of RSF and the continuous phase was fluorinated oil, as shown in Figure 6a.^49^ It was determined that not only does the ratio of the flow rate of the dispersed to the continuous phase affect droplet formation but also that protein concentration plays a crucial role on droplet size. The latter is due to the increase in viscosity with increasing protein concentration. Particles ranging from 2500 ± 110 nm down to 51 ± 6 nm could be systematically formed, and by utilizing the propensity of proteins to self-assemble, protein nanogels stabilized by supramolecular fibrils were generated (Figure 6b). The authors further showed the capability of such protein-based nanoparticles to act as cargo delivery vehicles using confocal microscopy (Figure 6c,d). The ability of these capsules to penetrate mammalian cell membranes and deliver intracellular cargo was demonstrated on ovarian cancer cells, where particles smaller than 200 nm could enter the cell via a phagocytosis mechanism, whereas particles that were 1000 nm could not pass through the membrane. Because of the biocompatibility and lack of toxicity, such natural protein-nanoparticles present excellent candidates for gene/drug delivery purposes and have advantageous characteristics for future biomedical and pharmaceutical applications.

Furthermore, the formation of double and higher-order emulsions, where droplet size can be precisely controlled and modulated is essential for tailoring release kinetics of active ingredients. To this effect, the systematic and reproducible formation of hierarchical emulsions has been extensively studied. In one of these studies, it was explored how different types of droplets can be encapsulated within an oil droplet resulting in a double emulsion with two aqueous internal droplets as can be seen in Figure 6e–g.^153^ Not only could droplet size be specifically controlled, but the number of internal droplets has also been regulated. Moreover, with the emergence of nonplanar (or 3D) microfluidics (schematic shown in Figure 6h), PDMS-based devices can also be used for generating hierarchical emulsions. In recent studies, biomolecular emulsions from protein solution have been formed. Monomeric protein solution was added as either the internal or middle phase, resulting in the formation of microgels surrounded by oil or core–shell nanofibrillar structures, respectively, following self-assembly.^154^ Here, not only was the number of internal droplets controlled (Figure 6i), but perhaps more importantly, the size of the oil shell thickness could be regulated, which is crucial if one is to tailor release kinetics of molecules from the internal droplet to the outside environment.

Finally, another application of droplet microfluidics which is gaining attention due to its relevance in mimicking cell systems is water-in-water emulsions. These all-aqueous emulsions allow for the biocompatible storage and processing of biomacromolecules which are extremely unstable due to their low interfacial tension. In order to stabilize such systems, lysozyme nanofibrils have been shown to effectively act as surfactants and adsorbed on the droplet interface, resulting in a cross-linked network of nanofibrils to generate colloidosomes.^155,156^ These “fibrillosomes” have been shown to exhibit multilayer deposition of fibrils which consequently allows for control over the capsule shell thickness and is thus ideal for regulating release of active molecular ingredients. Moreover, because of the lack of an oil phase in these systems, no de-emulsification process is required, making all-aqueous emulsions prime candidates for biological and pharmaceutical uses.

## Protein Condensates and Active Materials

**Figure 7:**
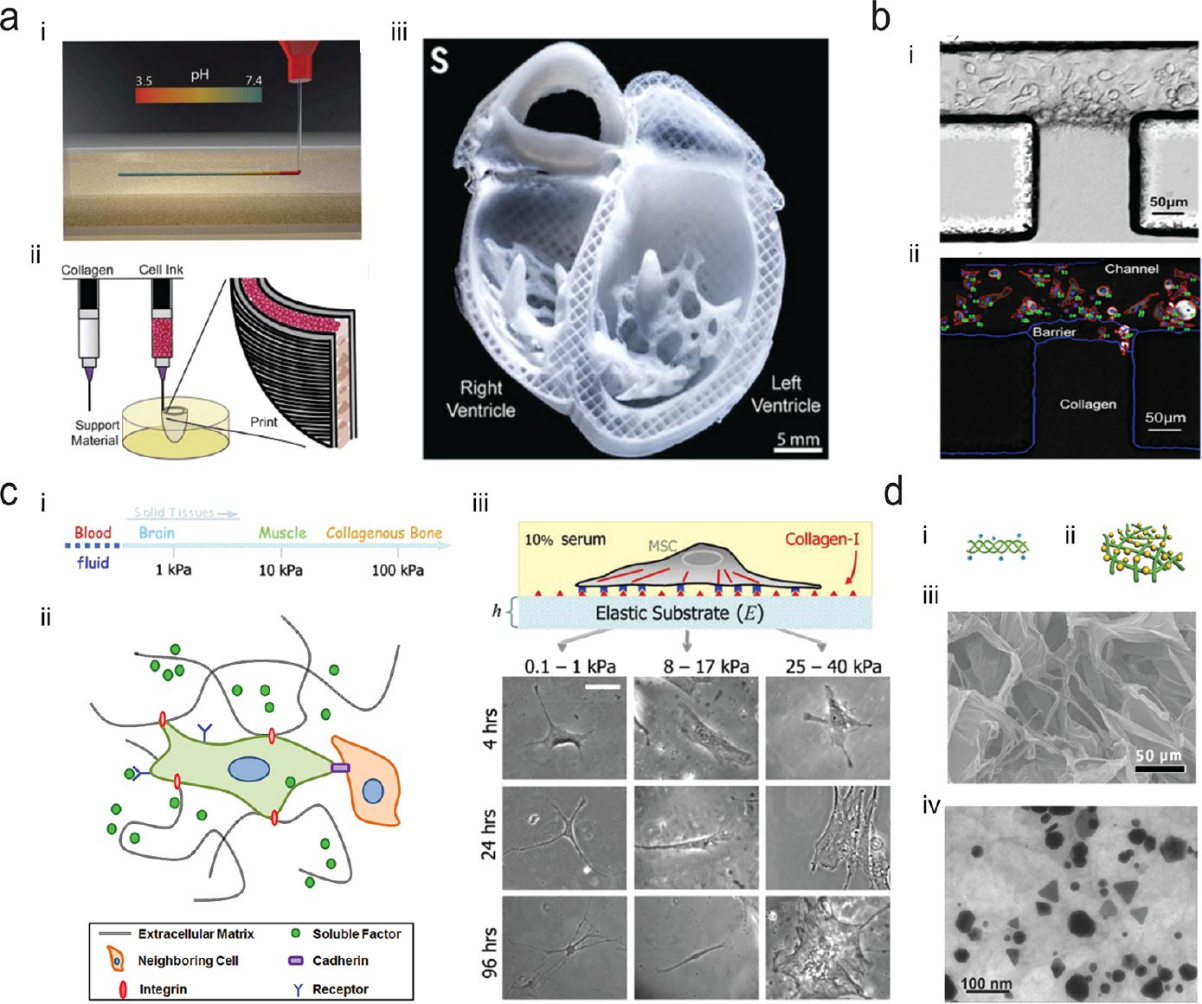
(a) 3D construction of collagen to rebuild the components of human hearts. i, Schematic of printing acidified collagen solution into neutral support bath (pH 7.4). ii, Schematic of dual-material printing using a collagen ink and a high-concentration cell ink. iii, Cross-sectional view of the collagen heart, showing left and right ventricles and interior structures. Adapted with permission from ref (46), Copyright 2019, The American Association for the Advancement of Science. (b) Transendothelial migration of cancer cells in a microfluidic system at the collagen-channel barrier. i, Brightfield image of collagen-channel system. ii, Fluorescent images of the segmentation of the projected cancer cell areas (red contours) identified by cell number in the microvessels. Adapted with permission under a Creative Commons CC BY license from ref (167), Copyright 2018, Springer Nature. (c) i, Native tissues demonstrate a wide range of stiffness. ii, Stem cells interact with extracellular matrices, neighboring cells, and soluble factors. iii, Stem cells differentiate into various cell lineages when cultured on collagen-coating substrates with varying elasticity. Adapted with permission from ref (170), Copyright 2012, American Chemical Society. (d) i, Schematic of a triple-helical structure of self-assembling collagen protein. ii, Schematic of a mineralized collagen hydrogel. iii, SEM image of a collagen-based hydrogel. iv, Transmission electron microscopy (TEM) image of a collagen hydrogel network containing gold nanoparticles. Adapted with permission from ref (160), Copyright 2016, Wiley-VCH.

The generation of mechanical forces by natural self-assembling systems has been found to play a key role in the formation of the nanoscale machinery of life. Thus, phenomena such as cellular movement and traction at surfaces have been found to be controlled through self-assembly of cytoskeleton proteins into well-ordered fibrillar structures.^157−159^ Collagen is one of the major components of the extracellular matrix (ECM) of mammalians.^160^ Fibers derived from collagen type I can distribute along various orientations in different tissues such as tendons, ligaments, and menisci.^161−165^ Collagen has been widely used as scaffolding materials for tissue engineering and regenerative medicine for tissues or organs such as nerves, bladders, bones, intestines, and so on.^166^ 3D printing and molds can assist in the construction of the collagen to rebuild components of the human organs, such as hearts (Figure 7a).^46^ Collagen has also been used in an extracellular matrix-integrated microfluidic chip as a microvessel, which promotes the understanding of the dynamics of cell–microenvironment interactions for cancer cell transmigration (Figure 7b).^167^ Cell–matrix interaction plays an important role in the control of the fate decisions of stem cells, for example, the cell–matrix distance, the deformation of the matrix, and the softness of the matrix can influence the differentiations of stem cells.^168,169^ Physical and chemical cues from the complex extracellular surroundings can also affect the self-renewal and differentiation of stem cells. Collagen and collagen-based materials have been used to mimic the complex and native microenvironment with a range of stiffness for 3D cell culture (Figure 7c).^170,171^ Injectable and self-healing collagen-based hydrogels have also been fabricated by electrostatic self-assembly and subsequent biomineralization for protein-based delivery methods and medical treatments (Figure 7d).^160^ Despite the broad interest in this class of materials, a detailed understanding of the relation between the molecular properties of nanofibrils, their nucleation and growth process, and their mechanical properties, specifically the force generated through their assembly, have remained challenging to explore through conventional techniques.

Through the development of low-volume approaches, such as the use of microfluidic techniques, exploring the dynamic properties of an active material such as amyloids’ proteins and peptide-based model systems have become available. Thus, recent studies have allowed expanding the current understanding of the forces generated by the polymerization process of well-characterized proteins such as actin^172^ to those produced by amyloid proteins and peptides.

**Figure 8:**
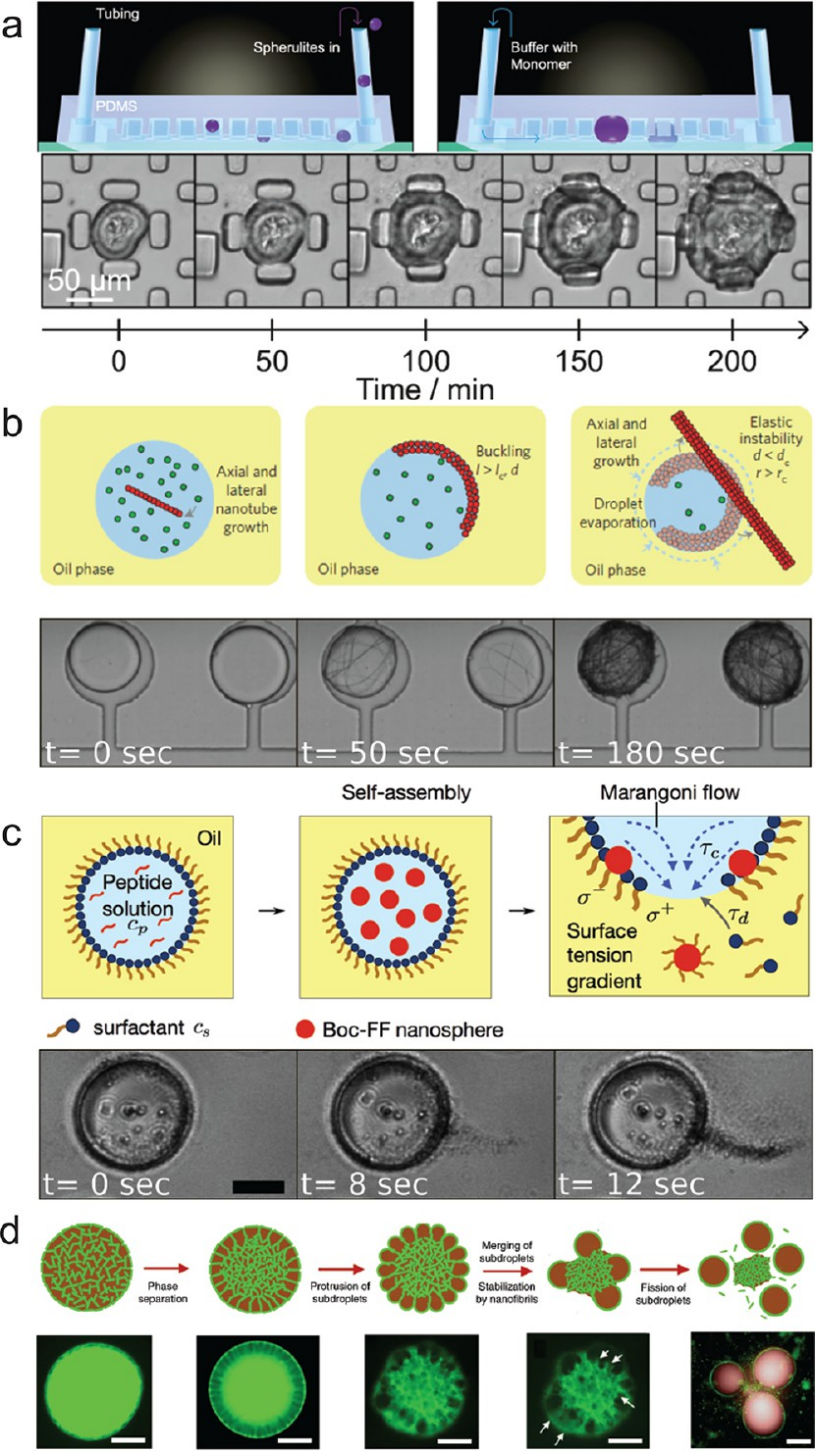
(a) Schematic diagram showing the introduction of preformed spherulites into the microfluidic device (top). To monitor the cantilever deflection, images were acquired through the glass slide at the bottom of the device (bottom). Reprinted with permission from ref (173). (b) Schematics of fibril growth within supercritical droplets and their buckling. Fibrils may pierce through the droplet because of an increase in their cross-section or through shrinkage of the droplet diameter (top). Reprinted with permission from ref (179). Copyright 2016, Springer Nature. Bright-field time-lapse microscopy of FF tube self-assembly and unbuckling due to droplet shrinkage (bottom). Reprinted with permission from ref (53), Copyright 2018, with permission from the Royal Society of Chemistry. (c) Scheme displaying the jet-like release of spheres through droplets and phase diagram indicating the condition for jetting (top). High magnification images of a single microdroplet releasing its nanosphere content during evaporation. Scale bar represents 30 μm. When the nanosphere assembly inside the droplet has reached a critical mass, nanosphere release could be detected within seconds (bottom). Reprinted with permission from ref (180), Copyright 2018, American Chemical Society. (d) Mechanism of budding-like division of w/w emulsion droplets mediated by protein nanofibrils. Schematic diagram (top) and fluorescence microscope images (bottom) describing the mechanistic steps in the budding-like division of w/w droplets. The fibril network (stained green) contracts and phase-separates from the remaining liquid phase through a dewetting transition. In this transition, the as-formed protrusions coalesce (as pinpointed by the white arrows) until a sufficient amount of fibrils adsorbs at the w/w interface to stabilize daughter droplets. Complete fission of dextran-rich subdroplets (faked red color) is observed after total decomposition of the fibril networks in the PEG-rich continuous phase. Scale bars, 100 μm. Figure reprinted with permission under a Creative Commons CC BY license from ref (156), Copyright 2018, Springer Nature.

Specifically, the force generated by the propagation of two amyloid proteins, insulin and lysozyme, has recently been determined.^173^ Through following the growth of these two protein aggregates in confined volumes, it was found that amyloid growth generates sufficient force to deform soft interfaces with moduli comparable to that of the cell membrane (Figure 8a).^173^ It has further been revealed that the forces generated by amyloid growth can reach the same order of magnitude as those resulting from the polymerization of cytoskeletal proteins. While cytoskeletal proteins have a limited scope for conformational change because of the need for reversibility, amyloid growth generates force through the formation of a maximum number of strong intermolecular interactions through the cross-β-sheet hydrogen bonding network^174^ and hence do not meet the dynamic requirements imposed on cytoskeletal protein self-assembly. Thus, the magnitude of force generation by amyloid growth highlights the potential and ultimate performance limits of protein-based active materials.

In a similar manner, the force originating from surface tension in microscopic droplets has recently been found to be sufficient to exert mechanical forces that can rearrange chromatin.^175,176^ Thus, intrinsically disordered proteins are also able to spontaneously form spatially well-defined compartments as a result of liquid–liquid phase separation (LLPS).^177^ This phase transition leads to the conversion of a homogeneous solution within the cytoplasm of the cell into dense liquid droplets and have also been shown to have the propensity to transform further into solid aggregated structures implicated in a range of neurodegenerative diseases.^38,178^ Specifically, the interplay between LLPS and chromatin is thus able to generate significant forces that can both push chromatin regions away from each other as well as bring them together.

Furthermore, a droplet-based microfluidic approach has recently been developed, and the transduction of chemical energy into mechanical work during fibril self-assembly has been explored using a minimalistic biomimetic amyloid system, the FF peptide.^179−181^ Through employing volume confinement by a microdroplet based strategy, the buildup of elastic energy as a result of peptide self-assembly, and its release within milliseconds was allowed. This work demonstrates a general phenomenon which may be applied to exploring the polymerization of amyloidogenic fibrillar structures and their suitability to transducing chemical energy involved in self-assembly into mechanical work. Recently, a similar microfluidic approach has been utilized to explore the ability of biomimetic amyloid short peptides to self-assemble into micron-scale colloidal particles and penetrate through an oil–water interface (Figure 8b).^180^ The self-assembly of Boc-FF nanospheres has been shown to be controlled using a microfluidic platform that encapsulates a reaction mixture on a chip, to form microdroplets. The peptide was observed to be able to undergo stable storing in a soluble state over several days. However, its rapid release could be triggered by inducing the self-assembly into nanospheres. The ability of the Boc-FF spheres to form jets while being released spontaneously from microdroplets indicates that this phenomenon involves a level of cooperativity and is not simply governed by a stochastic interface crossing by independent nanostructures, as the latter case would result in spatially isotropic rather than directional release. As mass transport of spheres is toward the region of release, this location must correspond to a zone of higher surface tension, suggesting that in this area surfactant molecules are depleted by the exiting spheres (Figure 8c). Moreover, this work demonstrates the ability of the Boc-FF spheres to act as carriers for other molecular species and transport them through the droplet interface. With a similar microdroplet formation approach, water-in-water emulsion were generated with protein nanofibrils enclosed (Figure 8d). The droplets were deformed and divided into budding-like subdroplets controlled by the phase transition of the protein nanofibrils network, which was driven by the change of its immersional wetting energy.^156^ Thus, modulating the supramolecular assembly state and phase behavior can control the transport of molecular species across interfaces and achieve the biomimetic cell division.

To conclude, by combining low-volume techniques together with theoretical studies, the ability of self-assembled amyloid systems to form ordered structures can now be expanded into exploring the forces produces by individual steps along the process.

## Translational Potential of Protein Materials

The self-assembly of protein molecules into supramolecular structures underpins the formation of many functional materials in nature, including spider silk and cytoskeletal filaments, as well as a number of common phenomena, such as the aggregation of proteins to bring structural functionality to food products such as yogurt and gelatin. It is well established that precisely controlling the self-assembly of proteins greatly contributes to exploiting and expanding their functionality in a wide range of applications. Recent progress toward controlling the self-assembly of proteins has shown potential for the development of structured proteinaceous materials for a wide range of consumer applications in areas such as human health, nutrition, food, or packaging.

However, the translation of lab-scale research into well-established commercial products is still limited, with only a few companies currently commercializing protein-based nanostructured materials. For example, the long-acting effect of FDA-approved gonadotropin-releasing hormone analogues Degarelix (Firmagon) and Ganirelix (Antagon) is attributed to their self-assembly into amyloid-like structures.^29^ Similarly, BluAct, a spin-off company from ETH (Zurich, Switzerland) commercializes protein–carbon hybrid membranes made from protein fibers and activated porous carbon that efficiently remove heavy metal ions and radioactive waste from water. These two examples clearly demonstrate the commercial applicability of nanostructured protein materials. Nonetheless, in order to apply these materials in a wider range of commercially viable applications, other parameters such as cost, regulation, and performance need to be considered.

There is a large number of studies that highlight the potential of nanostructured protein materials in a wide range of applications.^182^ However, such materials are often produced from lab-grade protein sources with a high degree of purity (such as lysozyme, insulin, etc.). The cost of producing such protein sources limits their use as feedstocks to supply for consumer applications that require large-scale volumes. In recent years, an increasing number of studies have shown the formation of protein self-assembled structures from traditional protein sources commonly found throughout the food and feed supply chain, such as proteins derived from the dairy industry. Protein fibrillar structures produced from whey protein or one of its main components BLG have shown potential in food applications,^183^ such as emulsion stabilization^184^ or enhanced iron bioavailability.^4^ However, there is an increasing demand to replace animal-based proteins for plant-based ones, not only due to their lower environmental impact but also due to their reduced cost (FAOSTAT 2011).^185^ While generation of fibril-like structures has been demonstrated in a variety of plant-protein sources, such as soy,^186−188^ pea,^77^ and zein,^189^ the conversion yields are generally lower compared with animal-derived proteins because of their inherent poor solubility in water and complex nature, and several purification steps are often required to remove insoluble protein fractions, thus preventing the process to be industrially scalable. It remains a challenge to optimize processes under which sustainable plant-based protein sources can be efficiently structured into fibrillar assemblies in a scalable manner.

## Conclusions and Outlook

In this Review, we have included recent studies on nanomaterial generation by using protein and peptide self-assemblies. We summarized the current techniques employed for the formation of bulk gel, films, fibers, micro/nanogels, condensates, and force generating active materials with nanofibrils. Furthermore, a wide range of techniques for generation protein biomaterials have been discussed.

We further link fundamental material science studies and their industrial applications and explain the potential of these materials in a wide range of areas, including drug delivery, tissue engineering, biosensors, environmental science, and food industries. In particular, nanofibril materials have recently become attractive for structural applications, given that they are among the most rigid proteinaceous structures. This is specifically relevant in the context of biodegradable materials to replace the use of oil-based polymers in packaging applications. Recent efforts to develop biobased alternative polymers, such as PLA, have enabled the transition from single-use oil-based plastics to biodegradable and compostable materials in many consumer products. However, most biobased plastics only degrade under industrial composting operations, and it still remains a challenge to develop a fully biodegradable material that provides a comparable performance.

In the future, by harnessing microfluidics, a toolkit can be developed to better understand the mechanical forces produced through the self-assembly of ordered fibrillar structures. This, in turn, can allow an increased understanding of the forces shaping *in vivo* systems, along with the development of bioinspired materials deriving their functionality from self-assembled fibrillar systems evolved by nature. Structured nanofibrillar protein materials could potentially provide comparable mechanical strength properties to the ones obtained using alternative biobased plastics. However, one of the main limitations of bioderived polymers such as proteins and polysaccharides is their low water-barrier properties, as opposed to conventional plastic materials. Through the development of composite-materials or the use of protein sources with a higher degree of hydrophobicity, the replacement of conventional flexible plastic packaging could be achieved in some applications.

## Vocabulary

**antiparallel β-sheet**: form of protein secondary structure where the polypeptide chains in the sheet run in opposite directions
**parallel β-sheet**: secondary structure element where the polypeptide chains are all oriented in the same direction
**regenerated silk fibroin (RSF)**: fibroin protein isolated from silk cocoons after resolubilization through lithium denaturation
**native silk fibroin (NSF)**: fibroin extracted directly from the gland of the silk worms
**protein condensates**: membraneless compartments consisting of a protein-rich phase inside the cytoplasm or nucleus of cells formed as a result of liquid–liquid phase separation
